# Comparative Study of Conventional Inverted ILM Flap Covering and ILM Flap Filling Technique in Idiopathic Macular Hole Treatment: A Meta-Analysis and Systematic Review

**DOI:** 10.1155/2022/4922616

**Published:** 2022-09-19

**Authors:** Mengying Tao, Guoqing Wang, Yueqin Gou, Ming Zhang

**Affiliations:** ^1^Department of Ophthalmology, West China Hospital of Sichuan University, Chengdu, China; ^2^Department of Neurosurgery, West China Hospital of Sichuan University, Chengdu, China

## Abstract

**Objective:**

This meta-analysis was performed to evaluate the anatomical efficacy and functional improvement of the conventional inverted internal limiting membrane (ILM), flap covering technique, and ILM flap filling technique for patients with idiopathic macular hole (MH).

**Methods:**

Literature from Pubmed, Embase, Cochrane Central Register of Controlled Trials, and Web of Science were comprehensively retrieved. The primary outcomes included the MH closure rate and postoperative best-corrected visual acuity (BCVA). The secondary outcomes were the proportion of external limiting membrane (ELM) and ellipsoid zone (EZ) defect recovery. Pooled odds ratios (ORs), weighted mean differences (WMDs), and 95% confidence intervals (CIs) were calculated using STATA 17.0 software.

**Results:**

7 studies that contained 139 eyes in the inverted ILM flap covering group and 121 eyes in the ILM flap filling group were selected. Pooled data suggested that the surgical treatment resulted in an overall MH closure rate of up to 97.12% (135/139 eyes) in the inverted ILM flap covering group and 99.17% (120/121 eyes) in the filling group, with no significant difference between the 2 groups (OR = 1.98, 95% CI: 0.55 to 7.09, and *P*=0.29). Similarly, the 2 techniques demonstrated equal effectiveness on the anatomical closure in MH with the average diameter smaller than 650 *μ*m (OR = 2.17, 95% CI: 0.48 to 9.77, and *P*=0.31) and larger than 650 *μ*m (OR = 1.58, 95% CI: 0.14 to 17.37, and *P*=0.71). However, compared with the filling technique, the inverted ILM flap covering technique was superior in postoperative BCVA (WMD = 0.11, 95% CI: 0.04 to 0.18, and *P*=0.0017) and presented a significantly higher proportion of reconstitution of ELM (OR = 0.02, 95% CI: 0.00 to 0.08, and *P* < 0.0001) and EZ (OR = 0.11, 95% CI: 0.04 to 0.32, and *P*=0.0001).

**Conclusion:**

The inverted ILM flap covering technique was associated with the superior reconstitution of outer layers of the retina, including ELM and EZ, and more improvement in postoperative BCVA than the ILM flap filling technique.

## 1. Introduction

Macular hole (MH) is characterized by an anatomical defect of the neuroepithelial retina in the fovea and manifests impaired central vision and metamorphopsia as initial symptoms [1]. An idiopathic macular hole is the most common form and was first discussed in fundus observation by Gass in 1988 [2, 3]. Pars plana vitrectomy (PPV) with the internal limiting membrane (ILM) peeling and gas tamponade is considered the standard treatment for MH, leading to the anatomical success rate of MH up to 90% [4]. However, the closure rate of MH larger than 400 µm decreases as low as 50–80% [5, 6]. In 2010, MH with a diameter greater than 400 *μ*m came to be remedied when Michalewska et al. [7] introduced the inverted ILM flap technique as an effective treatment, showing excellent improvement both in the anatomic and functional outcomes of vitrectomy.

In the conventional ILM flap procedure, known as “inverted ILM flap covering,” the ILM is peeled circumferentially to the edge of the hole; after trimming the perimeter, the central remnant of ILM is massaged and then inverted on the hole to cover it [7]. Afterwards, modified surgical techniques such as ILM flap filling technology were applied for larger or persistent MH after multiple vitrectomies. This operation allows the ILM flap to be inserted into the hole as a plug to stuff it [8, 9]. The filled ILM flap serves as a scaffold for glial cells to proliferate and migrate to the bottom of the hole, which facilitates gigantic MH anatomical closure [9].

The ILM flap technique has displayed gratifying superiority in anatomical and functional recovery since it was innovatively described for the treatment of MH [10, 11]. Currently, diverse surgical methods based on the ILM flap technique have been proposed for different types of MH to increase the efficacy of management and reduce surgical complications [12–14]. Nevertheless, it remains controversial about which technology is the more advantageous treatment option for MH. A large number of studies suggested that the ILM flap filling technique achieved extraordinary anatomical outcomes compared to ILM flap covering in MH [15–16]. Others held that ILM flap covering and its variation showed statistically similar outcomes in large MH [8–17]. There are few comprehensive consensuses focused on whether the ILM flap filling technique possesses more favorable anatomical closure efficacy and visual outcome in comparison with the ILM flap covering. In this study, we conducted a comprehensive and systematic meta-analysis of available literature comparing PPV combined with ILM flap filling and PPV combined with ILM flap covering for the treatment of MH.

## 2. Methods

### 2.1. Search Strategy

This meta-analysis corresponded with the Cochrane Handbook for Systematic Reviews of Interventions [18] and Preferred Reporting Items for Systematic Reviews and Meta-Analysis (PRISMA) Statement (https://prisma-statement.org/) [19]. Electronic databases including Pubmed, Embase, Cochrane Central Register of Controlled Trials, and Web of Science were searched comprehensively until May 27, 2022. Relevant literature was searched using the following terms: “macular hole^*∗*^” OR “macular break^*∗*^” OR “MH” AND “inverted internal limiting membrane” OR “inverted ILM” OR “ILM covering” OR “ILM insertion” OR “ILM transplantation” OR “ILM transposition” OR “ILM plug.” No language restrictions were applied. The search results were imported into reference management software (EndNote X9, Thomson Reuters, New York, NY, USA) for careful screening. Two assessors (MYT and YQG) deleted duplicate documents, sifted through relevant reports, and reviewed the remaining studies by reading the full text of the literature independently. Besides, their reference lists were also searched for available literature.

### 2.2. Inclusion and Exclusion Criteria of Literature

The literature according to the following criteria were included: (1) patients diagnosed with IMH, (2) intervention measure was technique containing the inverted ILM flap covering and ILM flap filling procedure, (3) one of the following outcomes should be reported, including the anatomical hole closure rate (according to optical coherence tomography (OCT) detection), or preoperative and postoperative best-corrected visual acuity (BCVA) expressed as the logarithm of the minimal angle of resolution (logMAR), and (4) study design such as prospective randomized controlled trials (RCTs), prospective non-RCT, and retrospective comparative studies were included. Exclusion criteria were as follows: (1) patients complicated with macular retinoschisis, retinal detachment, ocular inflammation, and history of vitrectomy, (2) studies without ILM flap covering or ILM flap filling group, (3) the data were not available for meta-analysis, and (4) case reports, surgical technique, and review literature.

### 2.3. Literature Data Extraction

Two investigators (MYT and GQW), respectively, screened the literature and extracted relevant information manually or by the semiautomated extraction tool WebPlotDigitizer (version 4.5; Pacifica, California, USA; https://automeris.io/WebPlotDigitizer) [20]. The available information involved the following clinical data: first author, year of publication, study country, study design, cohort size, mean age, gender ratio, and detailed disease information, including the diameter of MH, surgical procedure, preoperative BCVA, and follow-up period. The main outcomes were the MH closure rate and postoperative BCVA, while the secondary outcomes were the proportion of external limiting membrane (ELM) and ellipsoid zone (EZ) defect recovery. Disagreements were resolved via discussion with another author (YQG).

### 2.4. Quality Assessment

The quality of the selected RCT studies was assessed by using the Cochrane risk of bias assessment tool [21]. Six domains were assessed as follows: (1) selection bias, (2) performance bias, (3) attrition bias, (4) detection bias, (5) reporting bias, and (6) other potential biases. According to the instructions provided, each domain was classified into “low risk of bias,” “high risk of bias,” and “unclear risk of bias.” The quality of the included non-RCT studies was assessed based on the Newcastle-Ottawa Scale (NOS) [22]. Qualitative assessment according to three criteria were as follows: (1) sample selection, (2) comparability, and (3) exposure. One or two points were given to each item when it met the relevant criterion. In this article, the NOS scores of clinical trials were defined as low, moderate, and high quality, with scores of 1 to 3, 4 to 6, and 7 to 9. Studies with NOS scores above 4 points were included in the final analysis. Two reviewers (MYT and GQW) independently evaluated the bias of relevant clinical trials, and discrepancies were resolved via discussion.

### 2.5. Statistical Analysis

All statistical analyses were conducted with Review Manager (version 5.4; Cochrane Collaboration, Oxford, UK) and STATA software (version 17.0; Stata Corp LP, College Station, TX, USA). The odds ratios (ORs) and 95% confidence intervals (CIs) were reported for dichotomous variables, while the weighted mean difference (WMD) with 95% CI was calculated for continuous variables such as BCVA. The inverse variance (IV) or the Mantel–Haenszel (MH) method was used to deal with the clinical statistics. *P* < 0.05 was considered to indicate statistical significance. Statistical heterogeneity between clinical trials was assessed by the *χ*^2^ test, with *P* > 0.05 and *I*^2^ < 50%, indicating no significant heterogeneity, and a fixed-effects model was performed to assess. Otherwise, a random-effects model was applied. Sensitivity analysis was utilized to verify the credibility of the statistical conclusion. Besides, the funnel plot, Begg's and Egger's correlation test were conducted for publication bias.

## 3. Results

### 3.1. Characteristics of Available Studies

**Figure 1:**
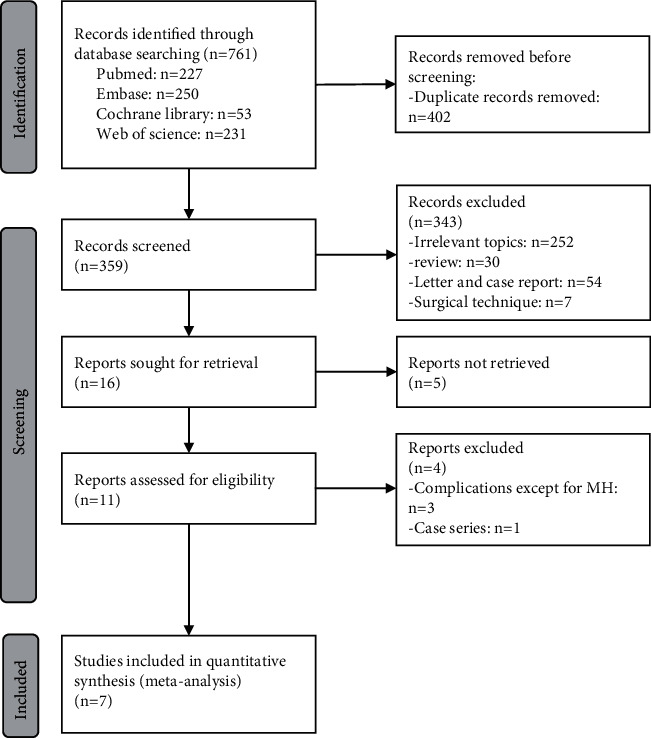
PRISMA flow diagram of the literature screening process.

The number of identified articles through database searching amounted to 761. Of these, 402 articles were duplicates, and 359 articles remained for preliminary review by reading the titles and abstracts. After further screening and retrieving, 343 articles were discarded for irrelevant topics, reviews, letters, case reports, surgical techniques, and infeasibility for retrieval. A total of 14 studies were assessed for eligibility by reading the full text. 4 reports were excluded, including 3 articles for complications except for MH and 1 record for case series. Subsequently, 7 studies (260 affected eyes) met the inclusion and exclusion criteria and were included in the final meta-analysis [8, 9, 15–17, 23, 24].  Figure 1 depicts the detailed study selection process. Among these studies, 2 reports [8, 15] were randomized controlled trials (RCTs) and 5 articles [9, 16, 17, 23, 24] were retrospective controlled studies, with a total of 139 eyes in the inverted ILM flap covering group and 121 eyes in the ILM flap filling group. The detailed characteristics are displayed in Table 1.

### 3.2. Outcomes

#### 3.2.1. MH Closure Rate

**Figure 2:**
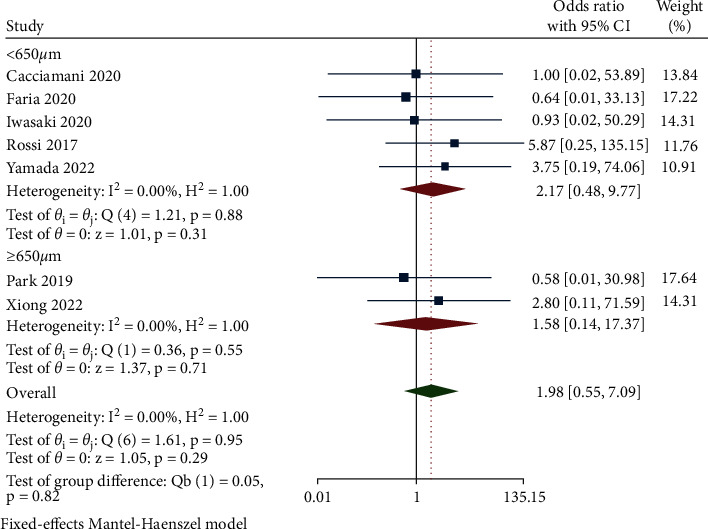
Forest plot of MH closure rate of ILM flap covering technique versus ILM flap filling technique.

The overall MH closure rate of the inverted ILM flap covering group and the ILM flap filling group was evaluated across 7 studies. With no heterogeneity observed between the studies (*I*^2^ = 0%, *P*=0.95), the fixed-effects model was applied for statistical analysis. The data showed that the ILM flap filling group possessed a better restorative tendency compared with the inverted ILM flap covering group, but the statistical difference was of no significance, with the anatomic success rate of up to 97.12% (135/139 closed eyes) in the inverted ILM flap covering group and 99.17% (120/121 closed eyes) in the ILM flap filling group (OR = 1.98, 95% CI: 0.55 to 7.09, and *P*=0.29) (Figure 2). Considering the influence of the diameter of the MH, the subgroup assessment based on 650 *μ*m as the reclassification standard of the MH size proposed by Ch'ng and his colleagues was carried out [25]. The pooled MH closure rate of ILM covering and filling treatment for MH with an average diameter smaller than 650 *μ*m (5 articles) was 96.39% and 98.73%, respectively. Similarly, the closure rate was 98.21% and 100.00% in the subgroup of MH larger than 650 *μ*m (2 articles), indicating an absence of significant difference in 2 subgroups (OR = 2.17, 95% CI: 0.48 to 9.77, and *P*=0.31 for average diameters smaller than 650 *μ*m and OR = 1.58, 95% CI: 0.14 to 17.37, and *P*=0.71 for average diameters over 650 *μ*m) (Figure 2).

#### 3.2.2. BCVA

**Figure 3:**
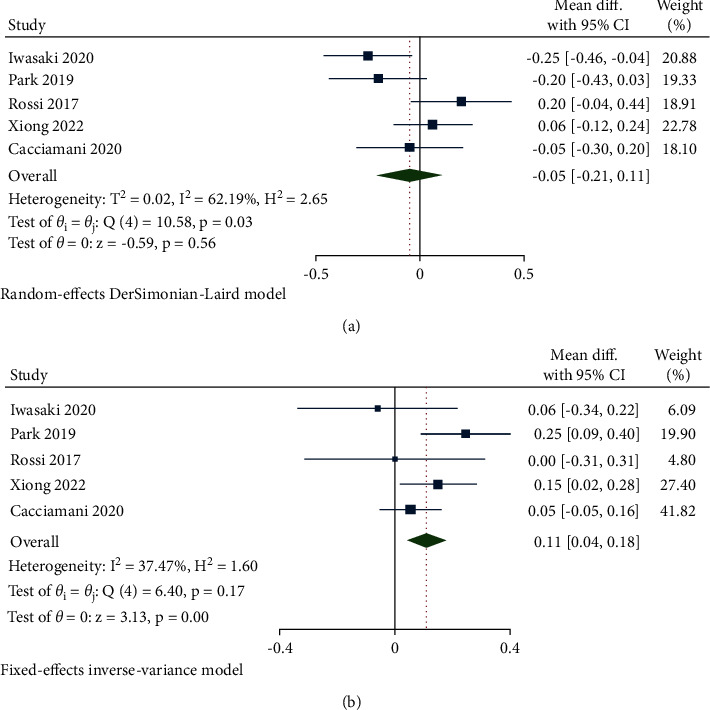
Forest plot of BCVA of ILM flap covering technique versus ILM flap filling technique. (a) Preoperative BCVA in logMAR. (b) Postoperative BCVA in logMAR.

5 studies reported preoperative and postoperative BCVA in a form suitable for meta-analysis. For the preoperative BCVA assessment, a random-effects model was used because of the great heterogeneity (*I*^2^ = 62.19%, *P*=0.03). The pooled data did not indicate any significant difference between the 2 groups (WMD = −0.05; 95% CI: −0.21 to 0.11, and *P*=0.56) (Figure 3). Nevertheless, after being merged through the fixed-effects model (*I*^2^ = 37.47% and *P*=0.17), the postoperative BCVA (WMD = 0.11, 95% CI: 0.04 to 0.18, and *P*=0.0017) in the inverted ILM flap covering group was significantly better when compared to the ILM flap filling group (Figure 3).

#### 3.2.3. ELM and EZ Defect Recovery Rate

**Figure 4:**
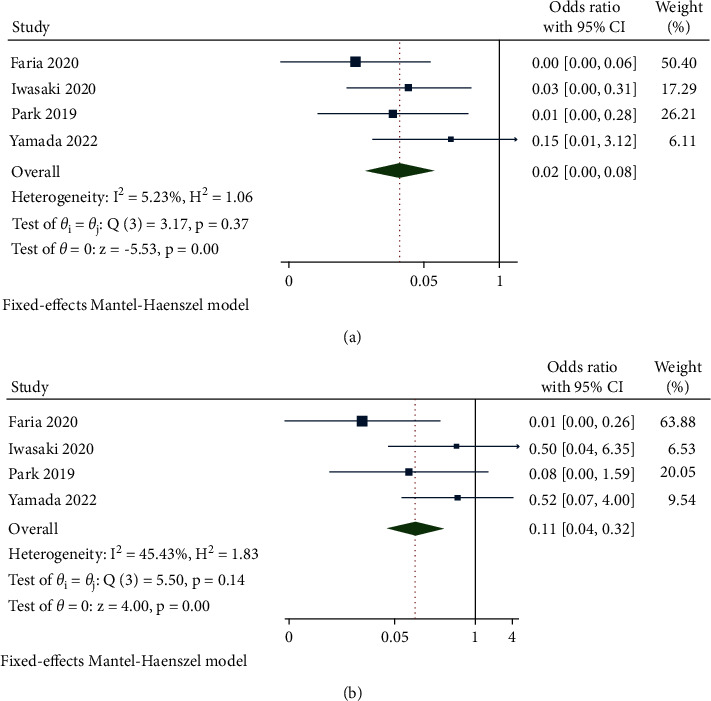
Forest plot of secondary outcomes of ILM flap covering technique versus ILM flap filling technique. (a) ELM reconnection rate. (b) EZ restoration rate.

A total of 4 studies reported the recovery rate of ELM and EZ defects after different therapies. The fixed-effects model was performed due to the moderate heterogeneity in the assessment of the rate of ELM recovery (*I*^2^ = 5.23% and *P*=0.37) and EZ reconnection (*I*^2^ = 45.43% and *P*=0.14). The respectively calculated OR was 0.02 (95% CI: 0.00 to 0.08) and 0.11 (95% CI: 0.04 to 0.32) for the proportion of ELM and EZ fully restored. The analysis demonstrated that the inverted ILM flap covering group was associated with a significant efficacy both in ELM and EZ complete recovery compared to the group treated with the ILM flap filling method (*P* < 0.0001 and *P*=0.0001, respectively) (Figure 4).

#### 3.2.4. Quality Evaluation, Publication Bias, and Sensitivity Assessment

**Figure 5:**
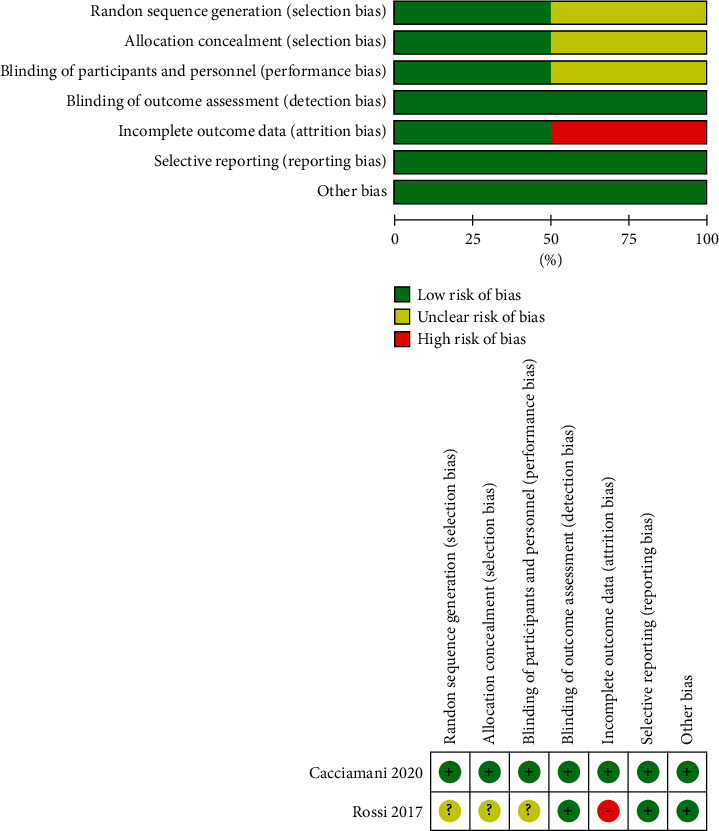
Evaluation of the bias in the included RCT studies by using the Cochrane risk of bias assessment tool. Green presents a low risk of bias, yellow presents an uncertain risk of bias, and red presents a high risk of bias.

**Figure 6:**
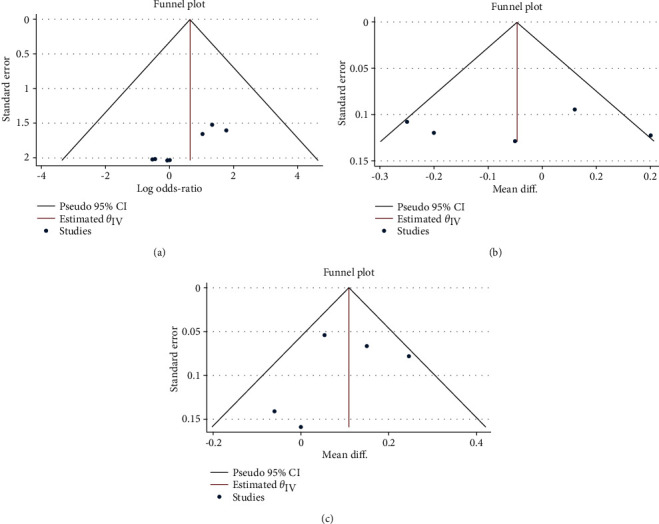
Funnel plot analysis of primary outcomes. (a) MH closure rate. (b) Preoperative BCVA in logMAR. (c) Postoperative BCVA in logMAR.

**Figure 7:**
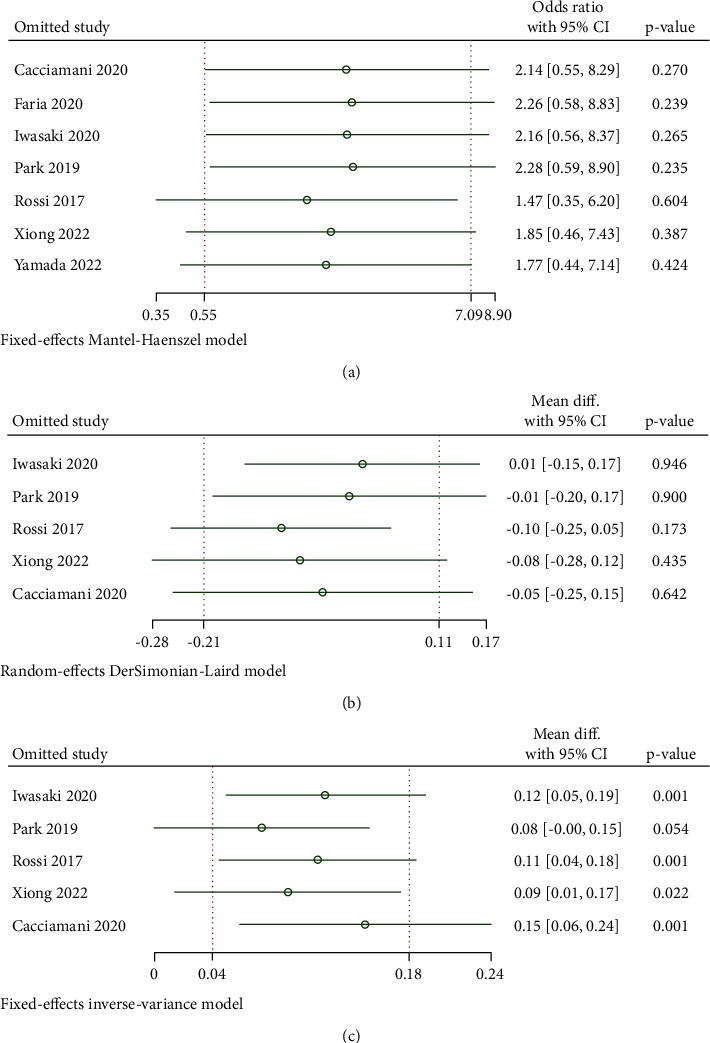
Sensitivity analysis, given named study is omitted. (a) MH closure rate. (b) Preoperative BCVA in logMAR. (c) Postoperative BCVA in logMAR.

The quality assessment of the 2 RCT studies was demonstrated based on the Cochrane risk of bias tool (Figure 5). 5 retrospective clinical trials were comprehensively evaluated by NOS (Table 2). Overall, the included studies were all at low risk of bias. Funnel plots were visually inspected to evaluate the publication bias of the anatomic success rate, preoperative, and postoperative BCVA. All scatter diagrams principally presented symmetrical points, which suggested that no obvious publication bias was visualized and the conclusions of the analysis were relatively credible (Figure 6). Begg's and Egger's correlation test did not detect significant publication bias as well. Specifically, the respective Begg's and Egger's test result was *P*=0.13 and *P*=0.23 for MH closure rate analysis, *P*=0.81 and *P*=0.98 for preoperative BCVA assessment, and *P*=0.81 and *P*=0.51 for postoperative BCVA evaluation. Outcomes of sensitivity analysis demonstrated the satisfactory stability of the calculated meta-analysis results, for which the new statistical results were not statistically different from the initial study results after erasing any one of the studies (Figure 7).

## 4. Discussion

As far as we know, this meta-analysis was the first to compare the anatomic and functional outcomes of the inverted ILM flap covering technique versus the ILM flap filling technique for MH. A total of 7 studies were combined for further analysis. Pooled data suggested that the 2 techniques resulted in similar anatomic success rates, while in the postoperative BCVA, the rate of reconstitution of ELM and EZ defects was much more favorable in the inverted ILM flap covering group.

It has been widely accepted that the anteroposterior and tangential traction in the vitreomacular interface contributed to the MH pathogenesis [26]. Traditional ILM peeling surgery assists in releasing the traction of the vitreous on the macula and flattening the retina to close the hole, which is insufficient for refractory MH with a large number of neuroepithelial defects [27]. ILM flap technique has been proven adequately effective for large MH in previous studies. Corresponding to earlier research, we found that those 2 surgical techniques manifested similarly extraordinary efficacy in repairing MH. The integrated MH closure rate was up to 97.12% in the inverted ILM flap covering group and 99.17% in the ILM flap filling group in this meta-analysis. A further subgroup assessment based on the average diameter of the hole revealed that the covering technique seemed to be as effective as the filling technique for the surgical treatment of eyes with different diameters of MH to achieve anatomical success.

In addition to the elimination of the macular traction, the ILM flap, no matter whether flap covering or filling, could act as a scaffold for glial cells to proliferate and migrate on the surface, thereby bridging the tissue dehiscence [7, 28]. Moreover, the residual Müller cells in the ILM flap could provide various cytokines to stimulate gliosis and relocate the photoreceptors, facilitating MH closure [7]. The ILM flap sealing the hole severs as a barrier, which separates the inside and outside of the hole to create a microenvironment encouraging RPE to transport fluids to the periphery of the hole [10, 29]. Theoretically, inserting ILM pieces into holes might be conducive to the restoration of larger MH, in the shape of an ILM sheet “plug,” thus providing a closed environment with utter volume restoration and long-lasting glial cell proliferation for the serious tissue defect [8, 30]. In contrast, we found that the covering technique seemed to be equal to the filling technique in terms of anatomical success in holes of different diameters, and our anatomical outcomes stressed the efficacy of both techniques for the surgical treatment of large MH.

However, it was still imprudent to conclude that both 2 surgical techniques performed equal effectiveness on MH over 650 *μ*m for only 2 included studies (98 eyes) in the subgroup that reported the surgical outcomes. Since a large number of studies reported the prompt and efficient reconstruction of the super large MH treated with the filling technique [3, 32], it is of great importance to conduct more comparative studies to explore the role of ILM flap filling technology in recovering giant neuroepithelial defects in the future.

Based on the results of the present analysis, the preoperative BCVA was of no significant difference between the 2 groups, which indicated that the preintervention effects were balanced and the postoperative BCVA was comparable. Besides, the improvement of postoperative BCVA in inverted ILM flap covering patients was better than that in patients with ILM flap filling. Except for Rossi et al.'s study, all 6 studies undoubtedly suggested that the covering technique obtained a clear superiority in visual gain in contrast to the filling technique. The result of visual prognosis is probably attributable to the reconstitution of the outer retina, including ELM and EZ, where photoreceptors align and reconnect with retinal neurons [33–36]. Equivalent to previous results, we found that BCVA after different procedures was consistent with the outcomes of complete recovery in ELM and EZ [37, 38]. This was also described by Rossi et al. [15]. They found that the outer retina persisted with cystic changes during the 3 months after the covering procedure. Instead, inserted ILM pieces disrupted the inner retinal layers while the photoreceptor layer seemed to evade additional intervention after the filling operation, leading to similar photoreceptor layer gaps and mid-term postoperative BCVA in the 2 groups [15]. In general, the covering ILM flap provides direct contact with the surface of the inner retinal layers, resulting in prompt reconstruction of the layer and subsequently following the restoration of the outer layers, without intervention to RPE and photoreceptors unless the ILM flap is out of position during the management [39]. Less glial proliferation along with much preferable outer retinal formation in patients treated with the covering technique has been reported in the majority of the included studies, which plays a crucial role in visual recovery [9].

However, there are several concerns related to the ILM flap filling technique. On the one hand, filling the ILM flap sheets into the hole might be associated with mechanical injury of RPE in operation. Moreover, the intrusive ILM flap could become an obstacle during the repair period, thus throwing the neurosensory retina rearrangement into disorder [17]. On the other hand, several chromovitrectomy vital dyes, including indocyanine green (ICG), trypan blue (TB), brilliant blue *G* (BBG), and triamcinolone acetonide (TA), have been reported to have potentially toxic effects on the retina [40–43]. The ILM flap pieces with residual dye consistently congest the hole and adjoin with RPE and neuroepithelium, enabling inevitable chemical damage to the retina. Those long-term mechanical barriers and chemical damage in the central fovea appear to be relevant to the development of poor visual outcomes and disorganization of the outer layers in patients who underwent the ILM filling technique. Nevertheless, we ought to be fully aware that a sufficient follow-up is essential to visual recovery regardless of the surgical technique, for the process is still challenging and permanent for large MH to achieve the restoration and rearrangement of photoreceptors.

There were several limitations demonstrated when explicating the results of this meta-analysis. First, the number of included studies providing available data was limited, and the inadequate quantity of accessible data affected the ultimate results. Second, the limited number of cases and relatively short follow-up of the patients resulted in a deficiency of additional information such as visual outcomes, recurrence rate, and operative complications in the long run. Therefore, we were not able to conduct a comprehensive analysis without these important outcome indicators. Third, the potential deviation resulting from a shortage of information about the duration of MH, vital dye, and the final endotamponade ought to be taken into consideration when the statistical analysis was performed. Last, converting the information on the graphs to statistical data might bring extra bias.

## 5. Conclusion

This meta-analysis provided evidence that the inverted ILM flap covering technique could contribute to the superior reconstitution of outer layers in the retina, including ELM and EZ, and consequently more improvement in postoperative BCVA than the ILM flap filling technique, although both obtained significantly high anatomical closure success of MH. However, more randomized and prospective studies with larger sample sizes and longer follow-up duration are necessary to confirm the efficacy of the inverted ILM flap covering technique.

## Figures and Tables

**Table 1:** Demographic characteristics and ophthalmic information of the included studies.

Study	Country	Study design	Group	No. eyes	Age (years)	Gender (male and female)	Duration of MH (months)	Minimum diameter of MH (*μ*m)	Staining	Endotamponade	MH closure rate (%)	Pre-BCVA (logMAR)	Post-BCVA (logMAR)	Follow-up time (months)
Rossi [15]	Italy	RCT	Covering	13	71.3 ± 9.2	5.8	<6	553 ± 164	BBG	20% SF6	84.6	1.17 ± 0.24	0.58 ± 0.39	3
			Filling	13	67.7 ± 7.3	6.7	<6	555 ± 153	BBG	20% SF6	100	1.37 ± 0.37	0.58 ± 0.42	3
Park [17]	South Korea	Retrospective	Covering	26	65.7 ± 7.2	3.23	7.1 ± 5.2	662 ± 121	BBG	18% SF6	100	1.20 ± 0.40	0.53 ± 0.20	6
			Filling	15	66.3 ± 7.8	6.9	4.1 ± 5.1	657 ± 122	BBG	18% SF6	100	1.00 ± 0.30	0.77 ± 0.30	6
Cacciamani [8]	Italy	RCT	Covering	14	N/A	7.7	<6	N/A	BBG	20% SF6	100	0.84 ± 0.32	0.79 ± 0.36	3
			Filling	14	N/A	5.9	<6	N/A	BBG	20% SF6	100	0.18 ± 0.12	0.23 ± 0.16	3
Faria [23]	Portugal	Retrospective	Covering	38	N/A	19.19	N/A	N/A	Brilliant peel dual	15% SF6	100	N/A	N/A	12
			Filling	24	N/A	16.8	N/A	N/A	Brilliant peel dual	15% SF6	100	N/A	N/A	12
Iwasaki [9]	Japan	Retrospective	Covering	13	64.3 ± 16.6	5.8	N/A	609 ± 111	BBG and TA	20% SF6	100	0.98 ± 0.30	0.61 ± 0.38	10.9 ± 4.1
			Filling	12	62.7 ± 17.9	3.9	N/A	582 ± 129	BBG and TA	20% SF6	100	0.73 ± 0.23	0.55 ± 0.32	16.2 ± 8.4
Yamada [24]	Japan	Retrospective	Covering	5	N/A	N/A	N/A	<400	N/A	SF6	80	N/A	N/A	12
			Filling	16	N/A	N/A	N/A	<400	N/A	SF6	93.8	N/A	N/A	12
Xiong [16]	China	Retrospective	Covering	30	64.2 ± 7.6	10.20	6.92 ± 11.41	1217 ± 186 (baseline)	ICG	Air	96.7	1.13 ± 0.32	0.77 ± 0.26	1
			Filling	27	66.6 ± 6.3	4.22	6.92 ± 11.41	1305 ± 188 (baseline)	ICG	Air	100	1.19 ± 0.39	0.92 ± 0.24	1

Data were converted into the form of statistical mean ± standard deviation as far as possible. MH = macular hole, Pre-BCVA = preoperative best-corrected visual acuity, post-BCVA = postoperative best-corrected visual acuity, LogMAR = the logarithm of the minimal angle of resolution, RCT = randomized controlled trial, BBG = brilliant blue *G*, TA = triamcinolone acetonide, ICG = indocyanine green, SF6 = sulphur hexafluoride, and N/A = not available for statistics.

**Table 2:** Newcastle-Ottawa scale for the retrospective studies.

Author	Selection				Comparability	Outcome			Total score
	1	2	3	4	1	1	2	3	
Park 2019	★	★	★	★	★	★	★	★	8
Faria 2020	★	★	★	★	★	—	★	★	7
Iwasaki 2020	★	★	★	★	★★	★	★	★	9
Yamada 2022	★	—	★	★	★	—	★	★	6
Xiong 2022	★	★	★	★	★★	★	—	★	8

Selection 1: representativeness of the exposed cohort, Selection 2: selection of the nonexposed cohort, Selection 3: ascertainment of exposure, and Selection 4: demonstration that outcome of interest was not present at the start of the study. Comparability 1: comparability of cohorts on the basis of the design or analysis. Outcome 1: assessment of outcome, Outcome 2: was follow-up long enough for outcomes to occur, and Outcome 3: adequacy of follow-up of cohorts.

## Data Availability

All data generated or analyzed in this study are accessible from the corresponding author for further inquiries.
